# Auditory learning through active engagement with sound: biological impact of community music lessons in at-risk children

**DOI:** 10.3389/fnins.2014.00351

**Published:** 2014-11-05

**Authors:** Nina Kraus, Jessica Slater, Elaine C. Thompson, Jane Hornickel, Dana L. Strait, Trent Nicol, Travis White-Schwoch

**Affiliations:** ^1^Auditory Neuroscience Laboratory, www.brainvolts.northwestern.edu, Northwestern UniversityEvanston, IL, USA; ^2^Department of Communication Sciences, Northwestern UniversityEvanston, IL, USA; ^3^Neuroscience Program, Northwestern UniversityEvanston, IL, USA; ^4^Department of Neurobiology and Physiology, Northwestern UniversityEvanston, IL, USA; ^5^Department of Otolaryngology, Northwestern UniversityChicago, IL, USA; ^6^Data Sense LLCChicago, IL, USA

**Keywords:** music training, neural plasticity, at-risk development, electrophysiology, reading, speech, community interventions, auditory learning

## Abstract

The young nervous system is primed for sensory learning, facilitating the acquisition of language and communication skills. Social and linguistic impoverishment can limit these learning opportunities, eventually leading to language-related challenges such as poor reading. Music training offers a promising auditory learning strategy by directing attention to meaningful acoustic elements of the soundscape. In light of evidence that music training improves auditory skills and their neural substrates, there are increasing efforts to enact community-based programs to provide music instruction to at-risk children. Harmony Project is a community foundation that has provided free music instruction to over 1000 children from Los Angeles gang-reduction zones over the past decade. We conducted an independent evaluation of biological effects of participating in Harmony Project by following a cohort of children for 1 year. Here we focus on a comparison between students who actively engaged with sound through instrumental music training *vs*. students who took music appreciation classes. All children began with an introductory music appreciation class, but midway through the year half of the children transitioned to the instrumental training. After the year of training, the children who actively engaged with sound through instrumental music training had faster and more robust neural processing of speech than the children who stayed in the music appreciation class, observed in neural responses to a speech sound /d/. The neurophysiological measures found to be enhanced in the instrumentally-trained children have been previously linked to reading ability, suggesting a gain in neural processes important for literacy stemming from active auditory learning. Despite intrinsic constraints on our study imposed by a community setting, these findings speak to the potential of active engagement with sound (i.e., music-making) to engender experience-dependent neuroplasticity and may inform the development of strategies for auditory learning.

## Introduction

The developing brain is hungry to engage with diverse and meaningful sensory input. Hearing sounds, and actively making meaning from those sounds, bootstraps language development, provides a framework for socioemotional bonding, and contributes to the development of auditory, as well as some non-auditory, cognitive skills (Kuhl, 2004; Kral and Eggermont, 2007; Conway et al., 2009; Kral and Sharma, 2012). Early acoustic experiences also play a formative role in guiding an individual's life of listening and learning, for better or worse. Although it has been clear for a long time that auditory deprivation, such as hearing loss, can hamper auditory development (Northern and Downs, 2002; Sharma et al., 2002; Roberts et al., 2004; Conway et al., 2009; Whitton and Polley, 2011), recent research has highlighted that a lack of *meaningful, effective, and consistent* auditory input can hurt as well—even in cases of normal hearing thresholds (Ahissar et al., 2006; Moore et al., 2010; Hornickel and Kraus, 2013). Moreover, animals reared in environmentally degraded or noisy environments, and those subjected to a transient hearing loss, have less precise auditory processing later life (Engineer et al., 2004; Zhou and Merzenich, 2008; Polley et al., 2013; Mowery et al., 2014).

Children who grow up in poverty have less developed language and cognitive skills than their peers, putatively reflecting some degree of linguistic deprivation (Bradley and Corwyn, 2002; Stevens et al., 2009). This is likely due to a confluence of factors, including greater environmental noise in low-income neighborhoods, poorer nutrition, and potentially fewer enriching auditory interactions with caregivers. As a group, these children have less precise neural processing of speech, creating special challenges for listening and learning. In particular, children whose mothers have lower levels of education than their peers have increased non-stimulus evoked activity in their neural responses (“neural noise”), an increase in trial-by-trial variability of their responses, and less robust encoding of spectral formant features in speech (Skoe et al., 2013a). Interestingly, children who grow up in poverty have particular deficiencies in neural processing that are evocative of those found in children with language based learning problems, which in some cases may be another variety of auditory impairment (Kraus et al., 1996; Temple et al., 2003; Sandak et al., 2004; Abrams et al., 2009; Anderson et al., 2010; Goswami, 2011; Chobert et al., 2012a; Conant et al., 2013).

In light of this evidence, there are increasing efforts to provide community interventions to at-risk youth that might counteract the lifelong challenges presented by poverty (Neville et al., 2013; Campbell et al., 2014). Central to these efforts is a principle of *actively engaging* children in their sensory environments to drive learning. However, there have been relatively few empirical evaluations of this tenet outside of the laboratory, presenting a roadblock to the development of effective strategies for auditory learning. One domain of attention has come from studies of music training, which has emerged as a potential direction for these efforts thanks to evidence that playing music augments auditory function and that these enhancements carry over to advantages for everyday communication (Gaser and Schlaug, 2003; Peretz and Zatorre, 2005; Magne et al., 2006; Kraus and Chandrasekaran, 2010; Chobert et al., 2011; Patel, 2011; Herholz and Zatorre, 2012; Strait and Kraus, 2014). Music training need not be lifelong to engender lasting improvements in the nervous system (Skoe and Kraus, 2012; White-Schwoch et al., 2013), and there is evidence that school-based programs initiated as late as high school can spark changes in auditory processing (Moreno et al., 2009, 2011; Chobert et al., 2012b; Tierney et al., 2013). Pragmatically, music training lends itself to large-scale community-based interventions due to the ease of providing simultaneous instruction to large groups (see Johnson et al., 2013). For example, El Sistema, the Venezuelan program, provides music instruction to hundreds of thousands of children annually (Majno, 2012). Moreover, there is mounting evidence that music training can also improve reading and its chief sub-skills, providing a practical benefit to music instruction for children both in and out of school (for review see Tierney and Kraus, 2014).

Harmony Project (Los Angeles, California) is a non-profit organization that has used a public health model to provide free music instruction to at-risk children from gang reduction zones for over a decade. Harmony's mission is to *promote* child growth and development, to *build* healthy communities, and to *develop* children as community citizens by providing students opportunities for free music instruction, appreciation, and performance (www.harmony-project.org). These children come from schools where ≥90% of students qualify for free or reduced lunch, reflecting the pernicious poverty that pervades the lives of children in this demographic^1^. Once a child is enrolled in Harmony Project, s/he receives free instruments, music instruction, and performance opportunities through high school. Harmony Project has enjoyed tremendous success and currently has 14 programs in Los Angeles, in addition to satellites in Ventura County (California), New Orleans (Louisiana), and Miami (Florida). According to its 2013–2014 annual report, Harmony enrolls >1600 students each year. From 2010 to 2014, 93% of graduating seniors matriculated to college, compared to 44% in Los Angeles County as a whole^2^ and in an internal survey >80% of Harmony Project parents reported that their children's grades, behavior, and mood had improved since enrollment.

Despite this promising evidence, it was unknown whether participation in Harmony Project confers improvements in nervous system functions important for listening and learning, similar to those reported in laboratory, classroom, and school-based studies of music training (Moreno et al., 2009, 2011; Strait et al., 2010, 2012; Chobert et al., 2012b; Skoe and Kraus, 2013; Tierney et al., 2013; Putkinen et al., 2014). Many of these functions are aspects of neural processing linked to reading abilities (Banai et al., 2009; Hornickel et al., 2009, 2012a; Hornickel and Kraus, 2013; White-Schwoch and Kraus, 2013; Kraus and Nicol, 2014), suggesting a potential for music training to generalize to literacy skills (Strait et al., 2011; Tierney and Kraus, 2014). Moreover, it was unknown which learning strategy would prove most efficacious in this population. On the one hand, music appreciation classes can engage students in elements of music training that might be beneficial for auditory function, such as timing and melodic sensitivity to auditory cues. From a pragmatic standpoint, this might lend itself to large group classes suitable for community engagement. But on the other hand, *active* music-making that integrates these elements may be critical for effective auditory learning that could improve communication skills.

Our laboratory was invited to conduct an independent investigation of the biological impact of participation in Harmony Project. Uniquely, this research program has allowed us to evaluate the effects of an *existing and successful community-based music program* instead of relying on one developed by scientists for the purposes of laboratory study. This unprecedented ecological validity allows us to put an established music program “to the test” vis-à-vis its outcomes for nervous system function. We have previously reported that 2 years' participation in Harmony Project enhances the neural distinction of consonant-vowel syllables, reflecting more precise neural encoding of fine consonant features (Kraus et al., 2014). Here, as a follow-up, we focused on a comparison of children who underwent a short course of active music training to those who only took Harmony's music appreciation class. We hypothesized that active engagement with sound is a key ingredient to engender biological changes in auditory processing. To test this hypothesis, we followed a cohort of children (ages 7–10 years old) as they enrolled in Harmony Project and measured neural responses to speech before and after an intervening year of training. Instead of our laboratory assigning children to training groups, Harmony staff followed their typical procedures unadulterated by our study activities. One group of children spent part of their time in *introductory music appreciation classes* and progressed to *group instrumental instruction* ~6 months later, as a function of ongoing programmatic constraints (e.g., availability of instruments and instructors, student progress, etc.; see Methods for details on the music training). The second group spent the entire year in music appreciation classes. We only determined each child's group membership after all data collection activities were completed. Motivated by evidence of the importance of active engagement during auditory learning (Ahissar and Hochstein, 1997; Polley et al., 2006; Wright et al., 2010; Anderson et al., 2013b), we predicted that we would observe biological changes only in the group that actively made music. In particular, we predicted that this group would have more precise neural encoding of formant features in consonants, namely, faster neural response latencies and more robust encoding of high frequency spectral features in speech—aspects of neural coding previously linked to both language skills (Banai et al., 2009) and music experience (Parbery-Clark et al., 2009a; Skoe and Kraus, 2013; Strait and Kraus, 2014).

## Methods

All study procedures were approved by the Institutional Review Board of Northwestern University. Legal guardians provided written consent for their children to participate. The subjects themselves provided written assent and were remunerated for their participation in study activities.

### Subjects

Nineteen students (12 female) in Harmony Project received perceptual, cognitive, and neurophysiological tests before (Year 1) and after (Year 2) their enrollment in Harmony Project. The children ranged from age 7 years 9 months to 10 years 2 months at Year 1 (mean, 9 years 1 month). Children were taken from Harmony Project's waitlist and guaranteed a seat in a Harmony Project class in exchange for enrolling in the study. Years of maternal education, as an estimate of socioeconomic status (Bradley and Corwyn, 2002), was collected from parents via a questionnaire.

### Group formation

Because a number of facilities and sites participate in Harmony project, there is some variation in how the music curriculum is applied. Consequently, the 19 participants naturally formed two groups based on these programmatic constraints. Our research team was blind to these assignments, and all testing was conducted at a central location provided by Harmony Project. The “Mus” group comprised 10 children who spent the entire year in the “Music appreciation” class (described below). The “Mus+Inst” comprised nine children who participated in the “Music appreciation” during the first half of the year and matriculated to “Instrumental” classes for the second half of the year, after an appropriate number of instruments became available at that project site and/or instructors judged the students to be ready to progress in the program. These children came from several Harmony Project sites, each of which faced its own constraints on instrument availability and used slightly different curricula; therefore, detailed information on what motivated each child's matriculation to instrumental training is not available. Students in the Mus+Inst group were given string instruments and participated in group music classes, sectionals, and in ensemble groups. These children received between 28 and 39 h of hands-on instrumental practice (plus home practice) over the course of the year, while children in group Mus had no hands-on instrumental experience beyond the basic recorder usage described below as a component of the Music appreciation class.

At Year 1, the two groups were matched on age, hearing thresholds, years of maternal education, and a variety of cognitive tests (see Table 1A). The two groups were similarly matched at Year 2 (see Table 1B).

**Table 1:** **Means (standard deviations) of behavioral measures for Group Mus+Inst and Mus at Year 1 and Year 2**.

**Measure**	**Mus+Inst**	**Mus**	***p*-value**
**(A) YEAR 1**			
Age (months)	109.11 (8.85)	109.56 (6.09)	0.903
Boys:Girls	1:8	6:4	0.027
Years of Maternal Education	12.89 (1.62)	10.89 (4.57)	0.233
Pure tone average (dB NHL)	4.03 (4.58)	7.92 (5.23)	0.113
WASI	96.89 (11.20)	101.78 (12.51)	0.395
TOWRE	104.89 (18.05)	101.33 (14.75)	0.653
**(B) YEAR 2**			
Age (months)	121.11 (9.20)	121.20 (5.85)	0.904
Pure tone average (dB NHL)	5.42 (6.70)	6.39 (8.25)	0.787
TOWRE	105.11 (16.38)	102.00 (15.86)	0.688

*The two groups were matched on age, years of maternal education, hearing, IQ, and reading fluency, before musical training. There was a greater proportion of girls in the Mus + Inst group (χ^2^ = 4.866, p = 0.027)*.

### Music training

Participants underwent Harmony Project's standard introductory curriculum. This music appreciation class met for 1 h, twice a week, covering fundamental pitch and rhythm skills, vocal performance, basics of improvisation and composition, and awareness of musical styles and notation. Basic recorder skills are also a part of this class. In some cases, depending on instrument availability and students' readiness, students progressed to instrumental instruction within this first year. The instrumental instruction was ~ 2 h/wk of group instrumental instruction with opportunities for ensemble practice and performance.

### Audiological tests

At Years 1 and 2, participants received an audiological screening. Air-conduction thresholds were measured bilaterally at octaves from 0.125 to 8 kHz (and interoctaves at 3 and 6 kHz), and all participants had thresholds ≤20 dB nHL, normal tympanograms (Type A), and normal distortion product otoacoustic emissions (DPs ≥6 dB above the noise floor from 1 to 4 kHz). The Year 1 pure tone average (PTA) of octaves from 500 to 4000 Hz was calculated for the right ear for group comparisons.

### Cognitive tests

Students were administered the Test of Word Reading Efficiency (TOWRE, Pearson) as a measure of reading fluency; the block design and similarities tests of the Wechsler Abbreviated Scale of Intelligence (WASI, Pearson) were used to generate a 2-scaled estimate of intelligence quotient (IQ).

### Neurophysiological testing & analysis

An Intelligent Hearing Systems SmartEP (Miami, FL, USA) system, equipped with the auditory brainstem response to complex sounds (“cABR”) research module, was used to record brainstem responses to a suprathreshold square-wave click and a 40 ms synthesized syllable /d/ (Banai et al., 2009; Skoe et al., 2013a). The /d/ contains an initial stop burst and rapid consonant transition; although it does not include a vowel portion, it is nevertheless perceived as a [da]. Both sounds were presented at 80 dB SPL to the right ear through electromagnetically-shielded insert earphones (ER-3As, Etymōtic Research, Elk Grove Village, IL, USA) and responses were digitized at 40 kHz. Rarefaction clicks were presented at 31.1 Hz and responses were filtered from 0.1 to 3 kHz. Latencies of waves I, III, and V were recorded and verified to be within clinically-normal limits (Hall III, 2006). Alternating-polarity /d/ syllables were presented at 10.9 Hz and responses were filtered from 0.1 to 1.5 kHz in Year 1 and from 0.05 to 3 kHz in Year 2. As this longitudinal project evolved, it was decided to open the response filters for the /d/ at Year 2 in order to capture a richer response. Consequently, no direct claims can be made about within-group improvements between years; however, group differences within a given year are valid for both years. Latencies of waves V, A, C, D, E, F, and O were recorded. Peaks were chosen on anonymized waveforms; all neurophysiological data were sent by the testing team back to Northwestern University where they were processed by a team blind to participant group. Peak latencies and amplitudes were extracted from the waveforms and the slope of the onset complex (peaks V and A) was calculated. Spectral amplitudes were calculated with a fast Fourier transform applied over a 20–42 ms time range with a 2 ms ramp on either side (Hanning window) using custom software written in MATLAB (The Mathworks, Natick, MA, USA). Spectral energy from 455 to 720 Hz was averaged to compute the middle harmonics, and from 720 to 1154 Hz for the high harmonics. This speech stimulus has been employed in several previous experiments and the neural response is well-stereotyped (Russo et al., 2004; Banai et al., 2009; Skoe and Kraus, 2013).

### Statistical analyses

Statistical analyses were performed in SPSS (IBM, Chicago, IL, USA). Due to the differences in filter settings between years, repeated measures analyses were not statistically appropriate. Instead we compared the two groups using multivariate ANOVAs at Years 1 and 2 separately. Another multivariate ANOVA was used to confirm matching between the groups at Year 1 on behavioral measures. A chi-square test was used to assess the balance of males and females between the two groups. Effect sizes reported are Cohen's *d*. Neural variables of interest included those previously linked to reading ability in children. These include all peak latencies (Banai et al., 2009), the slope of the VA peak complex (Wible et al., 2004), middle harmonics (Banai et al., 2009), and high harmonics (Hornickel et al., 2012a).

We discovered that the two groups differed on the latencies of the click-evoked wave V at Year 1, a measure of peripheral auditory function [*t*_(17)_ = 3.075, *p* = 0.007]. This is likely due to the higher proportion of females in group Mus+Inst (χ^2^ = 4.866, *p* = 0.027), for females are known to have faster neurophysiological responses than males (Jerger and Hall, 1980). To control for the sex differences in auditory neural response properties that may influence our speech-evoked measures (Krizman et al., 2012), in addition to potential individual differences in cochlear function that may affect response latency, we covaried for click-evoked wave V latency in all neurophysiological analyses.

## Results

As discussed above, group membership was not randomly assigned by the authors but was based on the natural progression of students through the Harmony program and programmatic constraints (e.g., instrument availability). Therefore, we investigated potential preexisting differences between students before undergoing musical training. A multivariate analysis of age, socio-economic status, hearing acuity, IQ, and reading fluency at Year 1 showed no differences between the two groups [*F*_(5, 12)_ = 1.065, *p* = 0.426]. None of the individual measures approached significance (see Table 1A). Additionally, the two groups did not differ in age or hearing thresholds at Year 2 [*F*_(3, 14)_ = 0.069, *p* = 0.976; see Table 1B].

**Figure 1:**
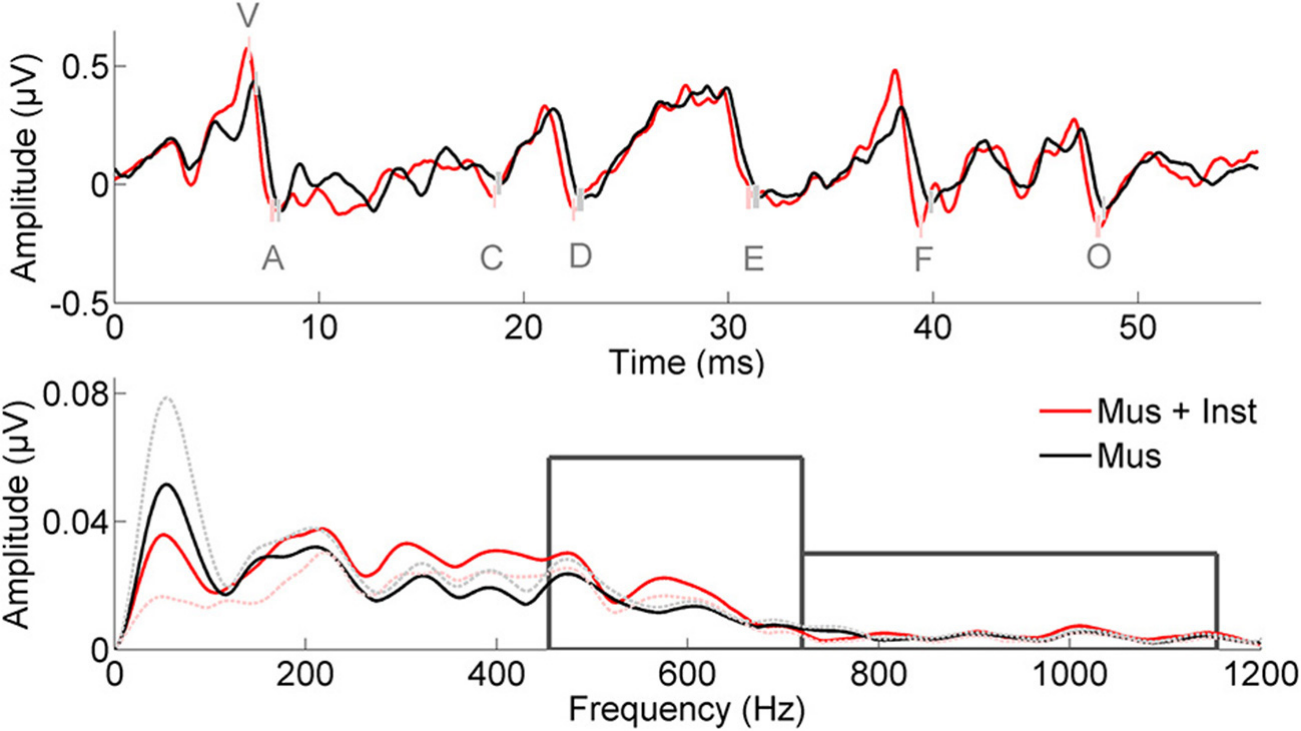
**Neural responses to the speech sound /d/ are presented in the time (Top) and frequency (Bottom) domains; grand averages are presented from children prior to training**. Response peaks of interest are labeled with a lettering system, and the small boxes illustrate standard errors between the two groups prior to training (Mus, black; Mus+Inst, red). When accounting for differences in click-evoked response latency, the two groups are matched on response timing. Boxes in the lower panel illustrate the two frequency domains of interest.

Similarly, we investigated potential differences between the groups in their speech-evoked brainstem responses before undergoing musical training (see Table 2A). A multivariate analysis of V, A, C, D, E, F, O, latencies, VA slope, and harmonics at Year 1 revealed no differences between the two groups prior to training [*F*_(10, 1)_ = 0.128, *p* = 0.981; see Figure 1]. No individual measures showed group differences, with all *p*-values greater than 0.106 (see Table 2A). This confirms that the groups did not differ in their speech-evoked responses before music training.

**Table 2:**
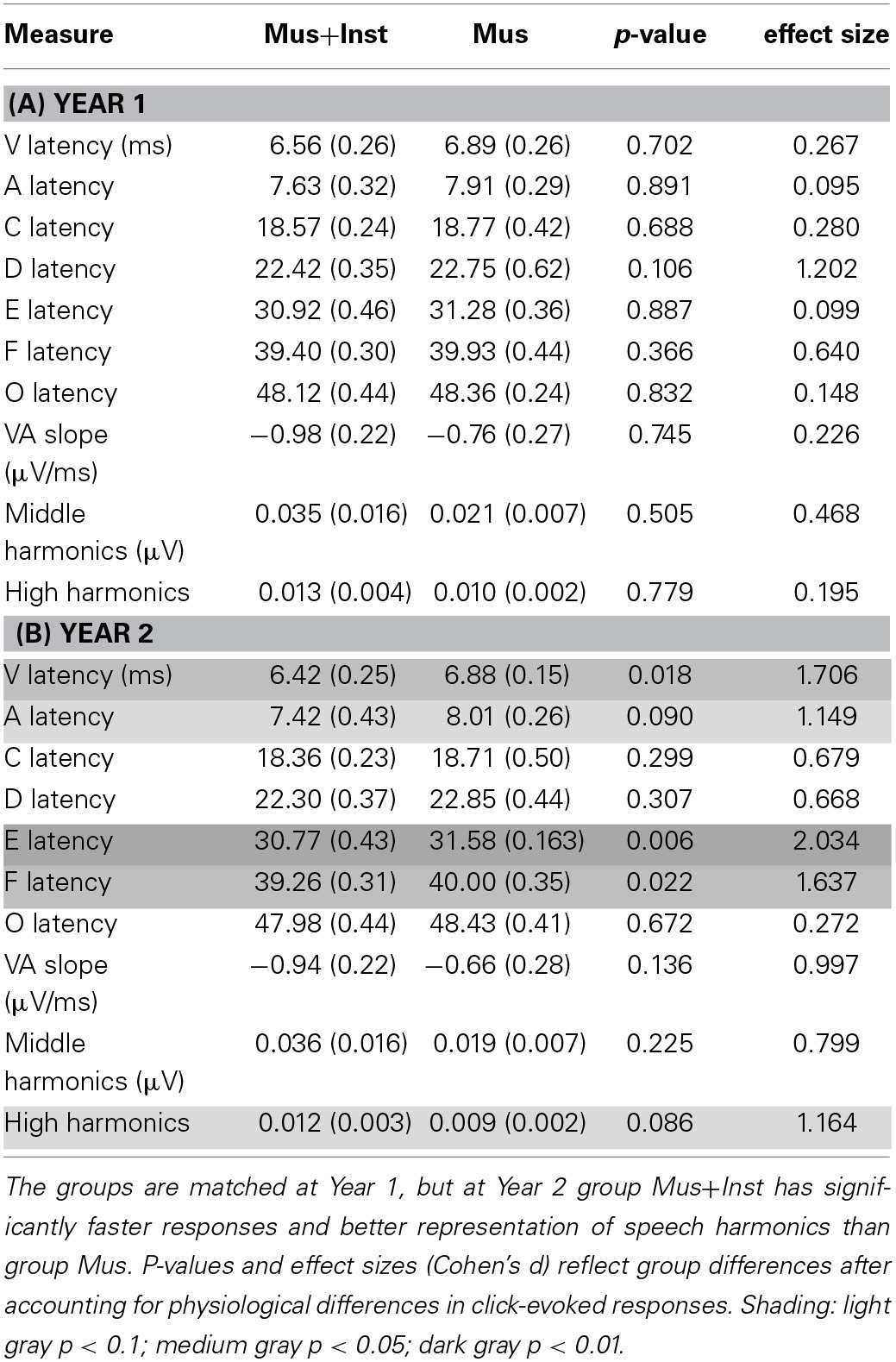
**Means (standard deviations) of neural measures at Year 1 (A) and Year 2 (B)**.

*The groups are matched at Year 1, but at Year 2 group Mus+Inst has significantly faster responses and better representation of speech harmonics than group Mus. P-values and effect sizes (Cohen's d) reflect group differences after accounting for physiological differences in click-evoked responses. Shading: light gray p < 0.1; medium gray p < 0.05; dark gray p < 0.01*.

**Figure 2:**
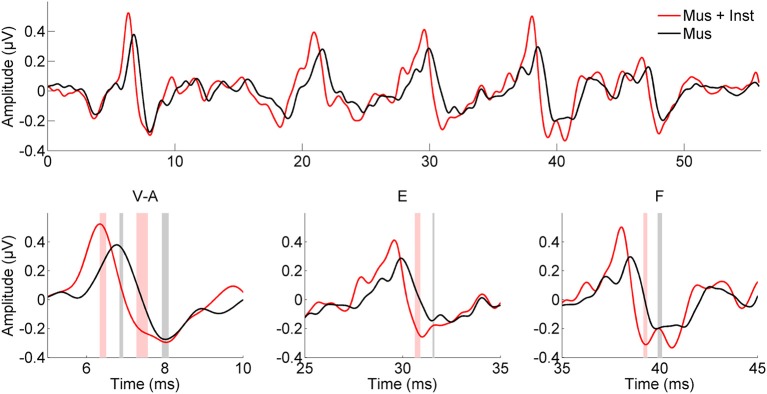
**Children who engaged in music appreciation and instrumental music training (Mus + Inst, red) have faster speech-evoked brainstem responses than do children who participated in music appreciation classes only (Mus, black)**. The particular aspects of the response that are faster in children with instrument training are those previously linked to reading ability (Banai et al., 2009). The shaded bars in the insets represent the mean peak timing ± 1 SE for each group.

**Figure 3:**
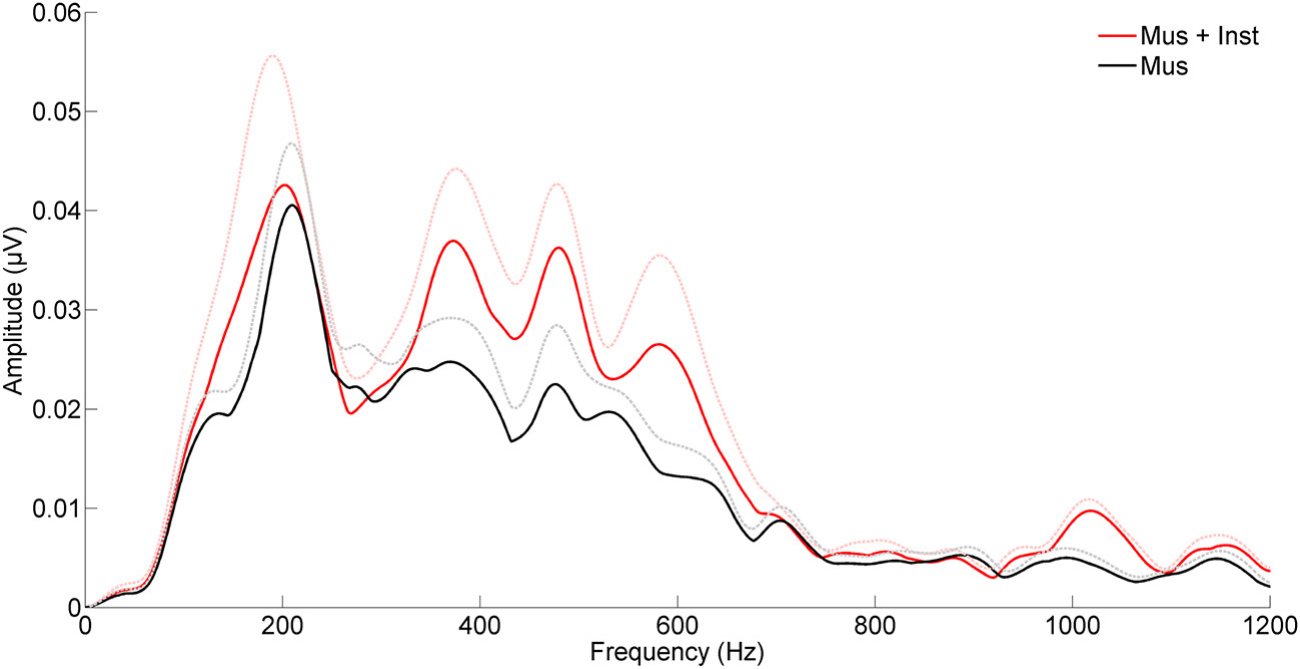
**Children who engaged in music appreciation and instrumental music training (Mus + Inst, red) have more robust brainstem representation of speech harmonics than children who participated in music appreciation classes only (Mus, black)**. The groups differ on harmonic ranges that have been previously linked to reading (Hornickel et al., 2012a). The hashed lines represent the mean + 1 SE for each group.

After undergoing a year of musical training, the children who participated in instrumental training showed faster and more robust brainstem responses to speech than children who participated only in music appreciation classes (see Table 2B). Although there were no group differences in reading fluency (*p* = 0.688, see Table 1B), participants in group Mus+Inst had faster response timing for peaks V [*F*_(1, 13)_ = 7.393, *p* = 0.018, Cohen's *d* = 1.706], E [*F*_(1, 13)_ = 10.511, *p* = 0.006, Cohen's *d* = 2.034], and F [*F*_(1, 13)_ = 6.811, *p* = 0.022, Cohen's *d* = 1.637] with a trending group effect for wave A [*F*_(1, 13)_ = 3.354, *p* = 0.090, Cohen's *d* = 1.149; see Figure 2]. Additionally, there was a trend for group Mus+Inst to have stronger representation of high harmonics [*F*_(1, 13)_ = 3.441, *p* = 0.086, Cohen's *d* = 1.164; see Figure 3]. Although group differences at waves A and for high harmonics were only trending, following Cohen's conventions these would still be considered “large” effects, placing the Mus+Inst group in the ≥90th percentile relative to the Mus group on these neurophysiological measures (Cohen, 1988).

## Discussion

Children who actively made music (in group instrumental lessons) after enrolling in Harmony Project had stronger neural processing of speech than their peers who only participated in a music appreciation class. This, to our knowledge, is the first comparison of instrumental music training *vs*. basic music appreciation classes in at-risk children in a community setting. Our results are a testament to the importance of active engagement with sound through its generation and online manipulation to drive auditory learning, especially through music (Kraus and Chandrasekaran, 2010; Patel, 2011). Uniquely, this investigation was in a community setting with an at-risk population and provides biological evidence as to these programs' potential.

Our group has delineated a series of “neural signatures”—constellations of enhancements and degradations to minute aspects of auditory processing—that reflect life experience and the quality of auditory processing at-large (Kraus and Nicol, 2014). Here, the musician signature has begun to emerge, reflecting a systemic change to a state of expertise—namely, faster neural coding of consonants and more robust encoding of high frequency spectral content in speech (as reviewed in Strait and Kraus, 2014). By engaging these children in repeated and positive interactions with sound, active music-making may have strengthened fundamental mechanisms of auditory processing, as has been reported in previous longitudinal studies of music training (Moreno et al., 2009; Chobert et al., 2012b). Although future work is needed with a larger sample size, better sex distribution, and more detailed information as to what guided students' progression through Harmony Project's curriculum (see below) these findings complement Harmony's success combatting the learning and social challenges their students face on a daily basis, and suggest the biological tractability of their programs. From a theoretical standpoint, our findings highlight the likely role active auditory engagement plays in auditory learning and by extension inform the development of strategies for learning in and out of the laboratory by emphasizing *active* auditory experience.

### Music, reading & auditory learning

Our finding fits well within a diverse series of longitudinal studies that have demonstrated the positive impact of music training on reading and its substrate skills in children this age (for review see Tierney and Kraus, 2014). These studies run the gamut from correlational studies (Peynircioglu et al., 2002; Trainor and Corrigall, 2010; Strait et al., 2011; Banai and Ahissar, 2013; Flaugnacco et al., 2014) to longitudinal experiments considering existing (Hurwitz et al., 1975; Rauscher and Hinton, 2011; Lorenzo et al., 2014) and experimenter-designed (Overy, 2000, 2003; Fisher, 2001; Gromko, 2005; Moreno et al., 2009; Bhide et al., 2013; Moritz et al., 2013) training regimens. By using an existing and successful community music training strategy, our findings fit with a growing movement toward investigations of ecologically-valid music training (Chobert et al., 2012b; Putkinen et al., 2013, 2014; Tierney et al., 2013). Uniquely, our findings present a conceptual advance by demonstrating that these training strategies affect the rapid neural processing of speech—processing that has been linked in previous investigations to language skills.

We show biological improvements in neural processes thought to be important for reading, demonstrated through a longstanding series of studies that have revealed correlations between auditory function and literacy skills (Tallal, 1980; Kraus et al., 1996; Tallal et al., 1996; Wright et al., 1997; Benasich and Tallal, 2002; Goswami et al., 2002; Ahissar et al., 2006; White-Schwoch and Kraus, 2013; Woodruff Carr et al., 2014). Children with learning problems (dyslexia, auditory processing disorder, language delays, and attention deficit hyperactivity disorder) have slower and less refined neural responses to the same speech stimulus used in the current study (Wible et al., 2004, 2005; Banai et al., 2009; Jafari et al., 2014; Malayeri et al., 2014). In fact, there is a systematic relationship between performance on reading tests and neural timing of the response to this speech sound (Banai et al., 2009). Therefore, although we do not show behavioral gains, it is encouraging that instrumental music training was associated with faster neural timing. It is important to keep in mind that the children in our study were, on average, normal readers (group average of ~100 on the TOWRE, which is the 50th percentile). Although there is room for improvement in our cohort, these are not disordered systems which might be especially prone to rapid behavioral recovery following intervention, as has been observed in longitudinal studies that found a potential for auditory training to generalize to reading skills (cf. Tallal et al., 1996; Hornickel et al., 2012b).

In addition, evidence from the auditory learning literature indicates that neurophysiological changes precede behavioral changes, suggesting that the biological enhancements we observe may eventually lead to salient outcomes for literacy (Tremblay et al., 1998; Ross et al., 2013). Here, we observe a neurophysiological effect of music training in about 6 months. Were we to follow these children for longer periods of time, behavioral advantages might emerge in the Mus+Inst group. Notably, we did not observe faster timing in the Mus+Inst group for all response peaks; in fact, we observed stronger differences as the processing demands of the /d/ stimulus increased; peaks E and F, for example, correspond to the neural encoding of the rapidly-changing consonant transition period, which is more difficult to process than peaks V and A (corresponding to the stop burst) (Tallal, 1980). Auditory learning, especially through music, may favorably improve the neural coding of behaviorally salient, yet acoustically complex, components of speech such as the consonant transition (Tallal et al., 1996; Russo et al., 2005; Song et al., 2008, 2012; Carcagno and Plack, 2011; Anderson et al., 2013a,b). During training, these salient-yet-complex elements of the soundscape may have been favorably enhanced for future automatic processing—but only under conditions of active auditory engagement through meaningful sound production and manipulation (Ahissar and Hochstein, 1997; Nahum et al., 2008; Strait et al., 2009, 2012).

Our interpretation is further motivated by complementary investigations of the overlap between neural systems that are deficient in poor readers, yet enhanced through music training. Whereas our techniques offer unique insight into very fast auditory processing (neural phaselocking exceeding 100 Hz), processing of slower phonemic features also carries this reading-music overlap. For example, a mismatch negativity study found that neural coding of voice onset time discrimination and syllable duration cues are deficient in children with dyslexia (Chobert et al., 2012a; see also Kraus et al., 1996). However, one year of active music training (adapted from the Kodály and Orff methods) improved these same neural functions (Chobert et al., 2012b; see also Zuk et al., 2013). The rich overlap between neural systems devoted to language and music likely underlies the benefits music training confers on reading, and its neural correlates across multiple timescales of auditory processing (Levitin and Menon, 2003; Patel, 2008; Kraus and Chandrasekaran, 2010). Besson et al. (2011) have argued that attentional and working memory networks, which are strengthened through music making (Parbery-Clark et al., 2009b; Strait et al., 2010; Kraus et al., 2012; Strait and Kraus, 2014), play a major role in this music-speech transfer over time. By engaging these networks in making sound-meaning connections, music can make important auditory cues behaviorally salient, affecting subsequent automatic auditory processing even when not making music.

### Providing enrichment to at-risk children

It is noteworthy that the children in whom we observed improvements in neural function are predominantly from low-income backgrounds. The majority of students from these children's schools qualifies for free or reduced lunch, a proxy that reflects an overall low-income level of a study body. Growing up in poverty has been linked to a series of biopsychosocial challenges, including for language learning and literacy. For example, children whose mothers have lower levels of education (another proxy for familial socioeconomic status) are estimated to have heard 30 million fewer words by age three than their peers, and at home to hear approximately two-fifths the number of *different* words per hour than their peers, reflecting a reduction in both the quantity and quality of linguistic experience (Hart and Risley, 2003; see also Fernald et al., 2013). A lack of linguistic input can stymie the development of literacy skills, eventually scaling to a large series of cognitive domains (Ritchie and Bates, 2013).

These behavioral challenges are also instantiated neurophysiologically (Noble et al., 2006; Raizada et al., 2008; Hackman et al., 2010). Just as our group has previously identified neural signatures of music training and reading ability, there is a signature biological impact of poverty on auditory processing (as indexed by maternal education; Skoe et al., 2013a). Children from lower socioeconomic backgrounds have less consistent and noisier responses to speech. Germane to the current study, these children also have less precise representation of formant features in response to the same /d/ sound used here. It is therefore encouraging to find that improvements in these biological domains are associated with instrumental music training in an at-risk population. In this regard, our findings complement a burgeoning line of research considering the promise of community interventions such as Head Start to stanch the health disparities caused by growing up in poverty (Neville et al., 2013; Campbell et al., 2014; Lorenzo et al., 2014; Zhu et al., 2014).

### Active engagement with sound: making music matters

Here we provide biological evidence that actively making music matters. This, to our knowledge, is one of the first demonstrations of the importance of active music practice in effecting changes in neurophysiology in a community setting with at-risk children. Active training was associated with faster neural timing and more robust neural encoding of speech harmonics relative to music appreciation classes alone. This comparison of active *vs*. academic in a community setting stands against the backdrop of a large series of studies that have demonstrated the potential for active music making to engender changes in neurophysiological and behavioral functions during childhood. Many of these longitudinal studies have compared active music making to other forms of activity such as painting training (Moreno et al., 2009; Chobert et al., 2012b; François et al., 2013) and have revealed a role for active music making to change neural function. Taken together with these studies and more, it would appear that actively making music may have a unique potential to positively affect neural functions (Norton et al., 2005; Schlaug et al., 2005; Fujioka et al., 2006; Forgeard et al., 2008; Hyde et al., 2009; Ellis et al., 2013; Putkinen et al., 2013, 2014; Strait et al., 2013; Tierney et al., 2013; Kraus et al., 2014).

The distinction between *making* music and *studying* music is an important consideration in the development of strategies for learning, suggesting these strategies should emphasize active experience that integrates sensory, cognitive, and reward circuits. Children from our two groups did not differ demographically, and both cohorts were highly motivated to participate in Harmony Project. The key difference between our training groups was in the *type* and *engagement* of their auditory training. Evidence from animal models supports the view that active, cognitive engagement with training stimuli gates the potential for neuroplasticity, and guides the form said plasticity ultimately takes (Ahissar et al., 1992; Polley et al., 1999, 2006). With respect to music training, it would appear that playing an instrument might be a key ingredient for engendering neurophysiological changes.

While to our knowledge there have been no empirical considerations of instrumental *vs*. music appreciation training in a community setting, our findings are consistent with experiments that have compared a training group to an active control group. For example, Anderson et al. (2013b) evaluated the biological impact of computer training on older adults. Active computer training that directed attention to fast changing speech sounds led to faster neural timing in processing speech, whereas viewing educational films and answering questions about the content did not (see also Anderson et al., 2013a, 2014; Anguera et al., 2013). Germane to music training, Tierney et al. (2013) demonstrated that two years of high school music lessons (instrumental or choir practice) also improved neural timing in response to speech, but no changes were observed in an active control group that participated in intense physical education classes and military drills. Comparisons of musical training *vs*. visual arts-based (painting) training have also found neurophysiological and behavioral enhancements only following music (Moreno et al., 2009).

Taken together, these results demonstrate the importance of active engagement with sound to drive neural plasticity in the auditory system. In the context of community or co-curricular music programs, our results provide solid evidence as to the importance of actively engaging students in *making* music. Theoretically, this active engagement with sound may not only set the stage for more precise automatic sensory processing, but also to benefit more from future *passive* auditory experiences later on in life. For example, interspersing periods of perceptual training with passive stimulus exposure can boost end performance on basic auditory tasks (Wright et al., 2010; see also Molloy et al., 2012). Auditory training in statistical learning paradigms is biased by past auditory experience (Lew-Williams and Saffran, 2012), including music training (François et al., 2013; Skoe et al., 2013b) and bilingualism (Bartolotti et al., 2011). Finally, older adults with past music experience have enhanced neural function up to 50 years after music training has stopped; this enhancement may reflect a training-induced change in automatic auditory processing, such that listeners profit from passive sensory experiences to recapitulate and reinforce enhanced auditory function (White-Schwoch et al., 2013). Much like action video games (Green and Bavelier, 2012), music may therefore help an individual “learn to learn,” changing putatively passive auditory experiences into actively engaging training as listeners navigate the everyday sensory world.

### Concluding remarks & future directions

We show that children who underwent instrumental music training in a community setting had faster and more robust neural processing of rapid speech elements than peers who only participated in a general music appreciation class over a single intervening year. This provides new evidence as to the efficacy of community music programs to instill changes in neural processing and highlights the importance of active engagement with sound in driving experience-dependent neuroplasticity.

In a companion study, we found that 2 years of active music training in Harmony Project, but not one, resulted in enhanced neurophysiological distinction of the contrastive speech syllables [ba] and [ga] on the basis of their formant features (Kraus et al., 2014). Unlike the current findings, the syllable-differentiation analysis employed a randomized control design to assign children to enroll in music making or to wait a year; we only observed changes in children who underwent 2 years of music training. Here, we observe an effect on different aspects of auditory function after several months of music training, namely, faster responses and more robust encoding of formant features in a single sound /d/. Future work is needed to disambiguate these effects. It may be the case that an initial enhancement in consonant encoding occurs following training that eventually underlies the more precise distinction between contrastive syllables. Unfortunately, with our small sample sizes we were unable to match groups on our previous findings' neural metric, making it difficult to map out the time course of auditory learning with respect to community music training. A large-scale randomized control study with frequent neurophysiological assays is needed to delineate this time course of how music training influences auditory brain function.

Our study cohorts are relatively small and poorly balanced with respect to sex; this sample size cautions us against making definitive recommendations for music-based auditory training. Moreover, our students came from multiple Harmony Project sites and, regretfully, we were unable to collect detailed information from instructors on what motivated each child's placement in different classes and matriculation to instrumental training. Finally, we cannot conclusively rule out the possibility that the group differences following training were affected by the different recording parameters; however we think it unlikely that these would only affect the group that underwent music training. Our findings highlight the biological potential of these programs, but also highlight the intrinsic challenges of this kind of research. Follow-up studies are needed to better answer these questions, with larger cohorts, a passive control, and careful tracking of programmatic constraints and curricula. It is also of strong interest to thoroughly investigate whether and how these effects may generalize to other speech sounds (cf. Kraus et al., 2014) and, hopefully, to behavior.

Broadly speaking, our goal is to understand what happens in the real world—what biological changes may be attributed to music in a community setting, away from the laboratory. This collaboration was a unique opportunity to assess the impact of an enrichment program on the developing brain that has already proven its viability and sustainability outside the laboratory. This community setting imposed some challenges to our study implementation, and teaches lessons not only about the biological potential of music training, but also about enacting community-based studies. Nevertheless, we see our study as a proof-of-concept that demonstrates the potential of community-based interventions to support children growing up in at-risk conditions. When considered against the backdrop of Harmony Project's success and the constellation of independent investigations that have established the positive associations between music training and literacy, we generalize our findings to provide encouraging support for the promise of community and co-curricular music programs during childhood, especially for children who come from underserved backgrounds. In general, independent of literacy and auditory processing, these community programs offer children an avocation that can provide personal satisfaction and enjoyment for many years.

## Author contributions

Nina Kraus and Dana L. Strait designed the study; Jessica Slater, Elaine C. Thompson, and Dana L. Strait collected the data; Trent Nicol provided reagents and analytic techniques; Jane Hornickel analyzed the data; Nina Kraus, Jane Hornickel, Trent Nicol, and Travis White-Schwoch prepared the manuscript; all authors provided input on the interpretation of the results the final manuscript.

### Conflict of interest statement

The authors declare that the research was conducted in the absence of any commercial or financial relationships that could be construed as a potential conflict of interest.

## Footnotes

^1^The current United States requirements to qualify for reduced lunch stipulate that families earn ≤185% of the federally-defined poverty line and ≤130% to qualify for free lunch (http://www.fns.usda.gov/school-meals/income-eligibility-guidelines).

^2^http://www.cpec.ca.gov/StudentData/CaCGRCountyGraph.asp?County=19
